# The Role of Angiogenesis Factors in the Formation of Vascular Changes in Scleroderma by Assessment of the Concentrations of VEGF and sVEGFR2 in Blood Serum and Tear Fluid

**DOI:** 10.1155/2020/7649480

**Published:** 2020-01-24

**Authors:** Arleta Waszczykowska, Roman Goś, Elżbieta Waszczykowska, Bożena Dziankowska-Bartkowiak, Michał Podgórski, Piotr Jurowski

**Affiliations:** ^1^Department of Ophthalmology and Vision Rehabilitation, Medical University of Lodz, Zeromskiego 113, 90-549 Lodz, Poland; ^2^Department of Dermatology and Venerology, Medical University of Lodz, Hallera 1, 90-647 Lodz, Poland; ^3^Department of Diagnostic Imaging, Polish Mother's Memorial Hospital Research Institute, Rzgowska 281/289, 93-338 Lodz, Poland

## Abstract

Systemic sclerosis (SSc) is a connective tissue disorder characterized by tissue hypoxia, excessive fibrosis of skin and internal organs, and angiogenesis imbalance. The aim of the study was to evaluate in SSc patients the association between the retinal microcirculation disturbances and the presence of peripheral trophic changes and to determine the role of angiogenesis factors in the formation of vascular changes in scleroderma. Twenty-five SSc patients and 25 age- and sex-matched healthy controls were included to the study. Assay of vascular endothelial growth factor (VEGF) and soluble VEGF receptor-2 (sVEGFR-2) in blood serum and tears was done for all patients and controls using enzyme-linked immunosorbent assay. Retinal blood circulation was investigated with fluorescein angiography (FA) in the SSc patients only. In our research, proportion of mainly hypertensive patients presenting with a large spectrum of retinal microvascular lesions was 72%, while proportion of patients with skin microvascular lesions within distal phalanxes of fingers and toes was 76%. We noticed that patients with pathological changes in the FA examination had finger ulcerations significantly more often than patients without changes in the eye fundus. There were no statistically significant differences in the serum concentration of VEGF and sVEGFR2 between subjects in both analyzed groups. Analysis of lower levels of VEGF (*p* = <0.001) and sVEGFR-2 (*p* = <0.001) in blood serum accompanied by simultaneous higher levels of VEGF/sVEGFR-2 ratio in tears of SSc patients, as compared with the control group, indicates the superiority of proangiogenic factors in patients' tears.

## 1. Introduction

Scleroderma, also known as systemic sclerosis (SSc), is a severe, chronic connective tissue disease. Complications of SSc may lead to insufficiency of many internal organs, including the eye. Systemic sclerosis can be divided into two basic categories: limited systemic sclerosis (lSSc), where skin hardening lesions does not exceed 1/3 of the forearm length and occurs also on the face and diffuse systemic sclerosis (dSSc) with generalized hardening [1].

SSc is characterized by three distinct pathologic processes: autoimmune inflammation, fibrosis, and angiogenesis. These abnormalities result in insufficient blood flow, causing severe tissue hypoxia [2].

Hypoxia is the main cause of new vessel formation. It is a complex process, regulated by many factors. The mutual ratio of stimulating and inhibiting angiogenesis factors determines its development and advancement. One of the important proangiogenic factors is vascular endothelial growth factor (VEGF). VEGF interacts with cells by stimulating VEGFR-1 membrane receptors (fms-like tyrosine kinase Flt-1), VEGFR-2 (fetal liver kinase-1 Flk, KDR) and VEGF-3 (Flt-4, fms-related tyrosine kinase 4) [2, 3]. In contrast, the soluble VEGF receptors sVEGFR-1 and sVEGFR-2 play a particularly important role in inhibiting angiogenesis. They bind the VEGF molecule before it reaches the receptor on the surface of the cells or bind directly to the membrane receptor, blocking VEGFR-1 and VEGFR-2. As a result, endothelial cells are not stimulated for migration and proliferation. Literature data indicate that of the two receptors, sVEGFR-2 has a more important functional role [4, 5].

The mechanisms that disrupt angiogenesis in the course of systemic sclerosis are not fully understood. Studies indicate that the chronic, uncontrolled activation of VEGF-A and sVEGFR-2 may play a significant role in the development of microangiopathy in the skin of SSc patients [6]. The range of peripheral microvascular lesions is believed to reflect the risk of internal organ involvement and overall disease assessment [7]. To date, no examinations assessing the effect of angiogenesis factor activity on retinal microcirculation disturbances in the course of systemic sclerosis have been described.

## 2. Aim of the Study

The aim of the study was to evaluate the association between the retinal microcirculation disturbances and the presence of peripheral trophic changes in patients with systemic sclerosis. It also determines the role of angiogenesis factors in the formation of vascular changes in the scleroderma by assessing the concentrations of VEGF and sVEGFR-2 in blood serum and tears.

## 3. Materials and Methods

### 3.1. Patient Selection

The study included a total of 25 systemic sclerosis patients: seventeen with lSSc and eight with dSSc. Diagnoses were based on the 1980 criteria of the American College of Rheumatology and the 2013 ACR/EULAR SSc Classification Criteria for SSc [8, 9]. Patients with limited systemic sclerosis (16 women and one man, aged 38-77 years) received vasodilating agents (calcium channel antagonists, benzodiazepines, or angiotensin receptor antagonists, sometimes together with pentoxifylline) and vitamin E. Patients with diffuse systemic sclerosis (eight women and one man, aged 35-70 years) received immunosuppressive therapy (low-dose corticosteroids-prednisone at 0.5 mg/kg bw/day) in monotherapy or in combination with a cytostatic (cyclophosphamide at 1.5 mg/kg bw/day), as well as vasodilating agents, similar to the lSSc patients.

The control group comprised 25 healthy age- and gender-matched individuals, who were excluded from the interview for chronic general diseases or ophthalmologic diseases or who were taking medication. Exclusion criteria for all study participants included active infections of the anterior segment of the eye, contact lens usage, ocular surgery in the previous six months, glaucoma, allergies, and usage of local ophthalmic drugs. The artificial tear preparations used by two patients with SSc were discontinued two weeks before the study.  Table 1 provides a detailed characteristics of the studied groups.

Consent to include clinical information in scientific studies was obtained from all the participating patients, following the tenets of the Helsinki declaration. In addition, local ethics committee approval was obtained from our institution for this investigation (nr RNN/332/06/KB).

### 3.2. Patient Examination

All the SSc patients were asked about the duration of Raynaud's phenomenon during history. The presence of trophic changes on the hand and foot fingertips was determined.

A thorough history of eye diseases was collected from all study participants.

Retinal blood circulation was investigated with fluorescein angiography (FA) in the SSc patients only. The investigation involved the use of fundus camera TRC 50 EX (Topcon TRC 50IX, Topcon Ltd., Tokyo, Japan) following pupil dilation with 1% tropicamide solution (Tropicamidum WZF Polfa 1%, Polfa Warszawa SA, Warsaw, Poland), and administration of 5 ml of 10% fluorescein solution (Fluorescite; Alcon Laboratories, Fort Worth, TX) into the basilic vein. The investigation was recorded for approx. 10 min.

### 3.3. Serum and Tear Collection

Venous blood and tear samples were collected from all participants between 8 am and 10 am. In order to obtain a sufficient volume of SSc patients' tears, they were collected during several sessions for consecutive days (mean 7 sessions; range 5-13 sessions).

The blood samples were immediately placed into individual Eppendorf tubes (Eppendorf, Fremont, Calif., USA) and centrifuged at 6,000 rpm, for five minutes at 4°C. The sera were stored at -80°C until used for the assay.

All nonstimulated tear samples were collected from the inferior lateral tear meniscus by using disposable 5 *μ*l microcapillaries (Microcaps; Drummond Scientific Co., Broomall, PA). The tear film meniscus on the bottom eyelid was visualized using a slit lamp ophthalmic microscope (SL-D2 Topcon Inc., Paramus, NJ, USA). Anesthetic drops were not instilled. After collection, the tear fluid was transferred into a 0.5 ml tube (Eppendorf, Fremont, Calif., USA) and subsequently recovered by centrifugation at 6,000 rpm at 4° C for 5 min and storage at –80° C until use.

### 3.4. Measurement of Total Plasma and Tear VEGF and sVEGFR-2 Concentration

Before analysis, the plasma and tears were thawed at room temperature. The total protein concentrations of VEGF and sVEGFR-2 were determined by commercially available ELISA methods (Human VEGF, Quantikine; R&D Systems, Inc., Minneapolis, MN, USA; Human sVEGFR-2/KDR/Flk-1, Quantikine; R&D Systems, Inc.) according to the manufacturer's instructions.

### 3.5. Statistical Analysis

In statistical analysis, continuous variables are presented as the mean ± standard deviation (SD). Normality of data distribution was checked with the Shapiro-Wilk. Frequencies of ulceration occurrence between groups were compared with Chi^2^ (with Fisher's exact correction). Differences between continuous variables were evaluated by means of the Mann–Whitney test if two groups were compared or the Kruskal-Wallis ANOVA by ranks with post hoc test if three groups were evaluated. Statistical analysis was performed using Statistica for Windows (version 12.0, StatSoft, Tulsa, OK, USA). A *p* value of ≤0.05 was considered significant.

## 4. Results

### 4.1. Patient Examination

**Figure 1:**
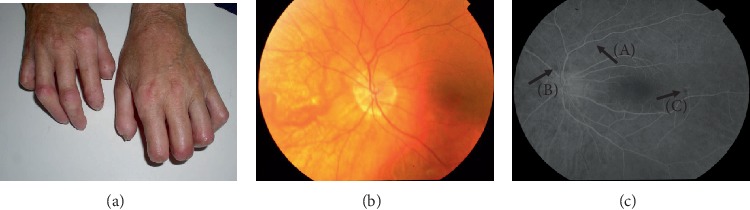
Three images of hands (a) and the eye fundus (b, c) of a patient, aged 51, diagnosed with systemic sclerosis six years ago. Vascular changes in the form of lysis of the distal phalanges of the hands fingers are visible (a). Color photo of the left eye fundus (B): sectional narrowing of retinal vessels (A), choroidal vessel transgression due to thinning of choriocapillaries and retinal pigment epithelium (B) can be observed. Results of fluorescein angiography of the left eye (c). Irregularities in the course and caliber of the retinal vessels (A) and foci of fluorescence blockade with local pigment epithelium regrouping (B, C) may be noted.

**Figure 2:**
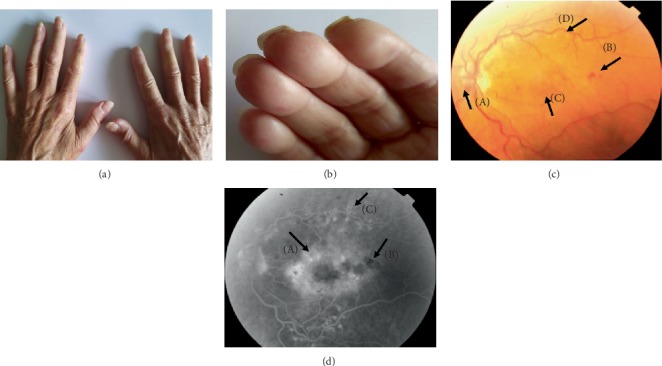
Panel of four images of hands (a, b) and eye fundus (c, d) of a patient, aged 61, diagnosed with systemic sclerosis seven years previously. Puffy fingers can be observed on both hands (a). Small scars from healed vascular ulcers within the second and third fingertips of the right hand at the edge of the nail (b). Color photo of the left eye fundus with ischemic retinopathy (c). Vasodilation of the optic disc (A), intraretinal petechiae (B), diffuse macular edema (C), and scars after laser therapy (D) may be noted. Results of fluorescein angiography of the left eye (d). A diffuse, poorly circumscribed and intensive hyperfluorescence of the whole posterior pole was observed in the recirculation phase; this was consistent with diffuse macular edema (A). Hypofluorescence foci (B) may be noted in the projection of extravasations and post-laser foci within temporal arcades (C).

Tables 1 and 2 indicate the duration of the Raynaud's symptom and the presence of trophic ulcers on the fingers or toes (Figures 1(a), 2(a), and 2(b)). No difference was observed in the frequency of occurrence of ulceration (*p* = 0.65) nor the duration of Raynaud's symptom (*p* = 0.37) between patients with limited (lSSc) and diffuse type of scleroderma (dSSc).

#### 4.1.1. Fundus Fluorescein Angiography of SSc Patients

The detailed characteristics of vascular changes of the eye fundus in patients with SSc are presented in Table 2. Fifteen patients demonstrated irregularities in the course and caliber of the retinal vessels. Stenosis and arteriosclerosis were observed in 15 patients. In 13 patients, these changes occurred on the background of hypertension. In one eye, the presence of the epiretinal membrane was found. Hard exudates in the posterior pole were noted in six eyes.

Dry age-related macular degeneration (AMD) was found in 14 eyes. Fluorescein angiography revealed the presence of hyperfluorescence foci, slightly increasing with time, without any leakage effect; these foci were consistent with soft drusen and window defects of retinal pigment epithelium.

Fluorescein angiography revealed delayed filling of choroidal lobules in 20 eyes of 10 SSc patients (three with dSSc and seven with lSSc). Among other retinal vascular abnormalities, occlusion of the peripheral arterial vessel was found in two patients with lSSc and presence of a sheathed venous vessel due to underlying inflammation in another eye; the latter finding was confirmed by fluorescein angiography. In three patients (one with dSSc and two with lSSc), thinning of choroidal capillaries and retinal pigment epithelium was noted (Figures 1(b) and 1(c)).

In the study group, in four eyes from two patients (one with dSSc and one with lSSc), ischemic areas surrounded by microaneurysms and intraretinal extravasation, dilatation of the vessel-free fovea region, and diffuse macular edema were observed. Peripherally on the fundus, areas lacking capillary perfusion and an enhanced capillary network were found (Figures 2(c) and 2(d)). Diabetes mellitus in these patients was excluded.

Patients with pathological changes in fluorescein angiography examination were more likely to demonstrate finger ulcerations than patients without changes in the eye fundus (*p* = 0.0173).

### 4.2. Blood Serum and Tear Concentration of VEGF and sVEGFR-2 in Patients with SSc and Control Group

**Figure 3:**
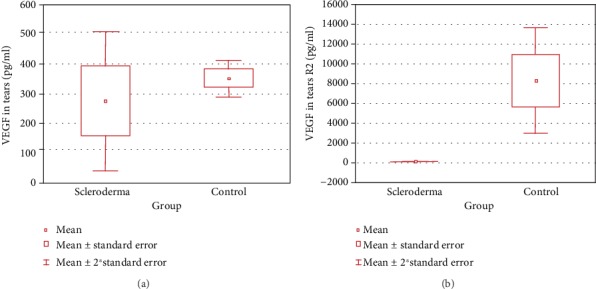
Box-and-whiskers plot of VEGF (a) and sVEGFR-2 (b) in patients with scleroderma and controls. Abbreviations: VEGF: vascular endothelial growth factor; sVEGFR-2: soluble form of vascular endothelial growth factor receptor 2; SD: standard deviation.

Statistical analysis revealed statistically insignificant differences in the serum concentration of VEGF and sVEGFR-2 between the two analyzed groups. In contrast, patients with systemic sclerosis demonstrated significantly lower VEGF and sVEGFR-2 concentrations in tears, compared to controls (both *p* < 0.001) (Table 3, Figure 3, box-and-whiskers plot of VEGF (a) and sVEGFR-2 (b) in patients with scleroderma and control).

**Figure 4:**
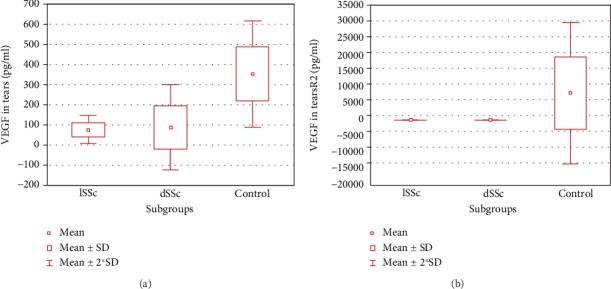
Box-and-whiskers plot of VEGF (a) and sVEGFR-2 (b) in patients with scleroderma subtypes and control. For the VEGF, three outliers were removed for the sake of plot clarity. Abbreviations: VEGF: vascular endothelial growth factor; sVEGFR-2: soluble form of vascular endothelial growth factor receptor 2; SD: standard deviation.

Mean concentrations of VEGF and sVEGFR-2 in the serum, when subtypes of scleroderma were concerned (lSSc or dSSc), did not differ significantly between groups (Table 4). However, significantly lower levels of VEGF and sVEGFR-2 were observed in the tears of patients with lSSc and dSSc in relation to controls (Table 4 and Figure 4).

The concentration of serum sVEGFR-2 was significantly lower in patients with dSSc and ulcerations than in those with dSSc but without ulcerations (9871.3 ± 405.6 vs. 12010.0 ± 261.6; *p* < 0.001). Serum VEGF levels followed a similar trend (237.7 ± 248.1 vs. 364.2 ± 29.7; dSSc patients with and without ulcerations, respectively); however, the difference was not significant (*p* = 0.1409). In patients with lSSc, no significant association was observed between ulcerations and VEGF parameters.

VEGF concentration was significantly higher in patients with closed peripheral arteries' lumen tears than in those without (1065.8 ± 1054.5 vs. 196.25 ± 196.3; with and without closure, respectively; *p* < 0.01). Also, the duration of Raynaud's symptom was significantly shorter in patients with ischemic retinopathy than in patients without (11.4 ± 5.7 vs. 5.5 ± 1.9; with and without retinopathy, respectively; *p* < 0.05).

The duration of Raynaud's symptom correlated negatively with serum VEGF levels (R = -0.54, p < 0.01).

## 5. Discussion

Our findings provide an insight into the relationship between the presence of vascular lesions within the retina and those of the skin in SSc patients. Of the patients included in the study, 72% presented with retinal microvascular lesions while 76% demonstrated skin microvascular lesions within distal phalanxes of fingers and toes. No significant differences in the incidence of retinal and skin microcirculation disorders were found between patients with either subtype of systemic sclerosis. However, our conclusions are tentative, as the spectrum of the retinal microvascular alterations observed was wide and heterogeneous, as many of these findings may be attributed to the high rate of hypertension among the participants and the important role of aging. In contrast, abnormalities in retinal fluorescein angiography were observed significantly more frequently in patients with concomitant lesions on the skin of the distal portions of upper and lower extremities.

Our results are inconsistent with results achieved in previous studies, which do not report any significant correlation between vascular lesions found in nailfold capillaroscopy and retinal vascular lesions: retinal microcirculation lesions were detected in approx. 34% of systemic sclerosis patients [10]. This difference may be attributed to the fact that 52% of the SSc patients in the present study suffered from concomitant hypertension, and the most commonly observed abnormalities included narrowed and tortuous retinal vessels. However, Aissopou et al. report that retinal vessel diameters observed in a group of SSc patients with concomitant arterial hypertension did not differ from those in the population of healthy individuals [11].

Our fluorescein angiography results revealed only few cases of patients with retinal microcirculation lesions specific for microangiopathy secondary to SSc; these were in the form of avascular zones in the choriocapillaris layer and a dilation of the capillary network. Our findings are consistent with literature reports [12].

One of the first symptoms in the course of systemic sclerosis includes lesions in blood vessels of skin and internal organs, manifested clinically as Raynaud's phenomenon. A relationship has been observed between the appearance of this phenomenon and blood vessel spasms within internal organs, such as the heart, kidneys, and lungs [13]. To date, no such correlation between the presence of Raynaud's phenomenon and ocular vascular lesions secondary to systemic sclerosis has been found [11], indicating that different mechanisms may be responsible for microcirculation disorders within the distal body parts and retina.

Although it seems natural that VEGF level should increase in response to tissue ischemia, this assumption is not obvious in the case of systemic sclerosis. Multiple authors have noted an increase [14], while others report a decrease [15] of serum VEGF level in SSc patients.

Our research did not reveal differences in mean serum VEGF and sVEGFR-2 between patients and controls, possibly due to the small number of study subjects. However, a negative correlation was observed between the duration of Raynaud's phenomenon and serum VEGF level. The presence of lower levels of VEGF and sVEGFR-2, together with an elevated VEGF/sVEGFR-2 ratio, in the tears of SSc patients compared with controls indicated the superiority of proangiogenic factors. Patients with lower levels of lacrimal VEGF were more likely to present choriocapillary atrophy or ischemic atrophy. Thus, it may be assumed that the presence of higher levels of VEGF in SSc patient tears may play a protective role in the eye. Other authors reported similar findings, demonstrating that decreased serum VEGF level was correlated with the presence of ulceration of phalanxes, and increased VEGF level accelerated the healing process [16, 17].

Despite the VEGF/sVEGFR-2 ratio being significantly higher in the limited scleroderma (lSSc) subgroup, lesions specific for ischemic retinopathy were observed in two SSc patients participating in our study: one lSSc and one dSSc.

It should be noted that compared to the mean values for the patient group, the lacrimal VEGF level was two-fold higher in two SSc patients and five-fold higher in two others. The VEGF/sVEGFR-2 ratio in these patients also was three- and seven-fold higher, respectively, than the mean value. In the eye fundus of these patients, only narrowing and tortuosity of blood vessels were found.

It should be noted that the increase of proangiogenic factor levels in SSc patients did not result in the development of proliferative retinopathy. Ozcelik et al. reported increased density of capillary network together with decreased VEGF level in gingival biopsies in SSc patients with chronic periodontal inflammation [15]. An explanation of these phenomena poses a challenge for modern medicine and would help in understanding, preventing, and inhibiting abnormal formation of new vessels also in other diseases.

## 6. Limitations

Data analysis should also take into account the fact that, due to the severity and chronic nature of the disease, almost all the patients examined took vasodilating agents before the study, some in combination with immunosuppressive agents.

Another limitation is that due to the rare occurrence of the disease, only a small number of SSc patients were included in the study. In addition, close cooperation was required with the patients and they needed to attend numerous visits to collect tear samples; several patients were concerned about potential complications and did not consent to intravenous contrast administration for fluorescein angiography examination. In two cases, patients were excluded from the study due to significant skin hardening and problems with palpation assessment of venous vessels.

The lack of nailfold capillaroscopy does not allow the SSc patients to be qualified according to early skin microvascular lesions. However, most of our SSc patients had fingertip ulcers or pitting scars caused by ischemia rather than an injury.

In addition, 52% of our patients also suffered from arterial hypertension.

## 7. Conclusion

Although we report a high incidence of various abnormalities in retinal microcirculation of patients with systemic sclerosis, our results should be interpreted with caution due to the concomitant effect of arterial hypertension, age, and inflammation. However, the role of fundoscopic examination in these patients is essential, providing invaluable information regarding SSc pathogenesis and treatment approaches.

Also the question why ischemic retinopathy develops only in some SSc patients irrespective of the stage of ischemic lesions of phalanxes remains to be answered, as well as whether the presence of retinopathy correlates with the stage of organ complications.

Long-term prospective studies are needed to extend and better understand the findings from this investigation.

## Figures and Tables

**Table 1:** Characteristic of analyzed groups.

Feature	Group			
	SSc (*n* = 25)	lSSc (*n* = 17)	dSSc (*n* = 8)	Control (*n* = 25)
Gender				
Female	21	14	7	20
Male	4	3	1	5
Age (years) (±SD)	57.1 (10.8)	60.2 (9.4)	50.6 (11.4)	59.4 (9.9)
Ulcerations				
Present	19	13	6	
Absent	6	4	2	
Raynaud time (years) (±SD)	10.9 (5.7)	11.4 (5.7)	9.9 (6.1)	
Gastrointestinal manifestation	15 (60%)	10 (59%)	5 (62%)	
Cardiac involvement	17 (68%)	11 (65%)	6 (75%)	
Pulmonary fibrosis	11 (44%)	7 (41%)	4 (50%)	
Renal abnormalities	4 (16%)	1 (6%)	3 (37%)	
Hematological involvement	6 (24%)	4 (23%)	2 (25%)	
Arthralgia	19 (76%)	13 (76%)	6 (75%)	

Results are shown as number and percent. Duration is presented in years as mean ± standard deviation of the mean. Abbreviations: SSc: scleroderma; lSSc: limited systemic sclerosis; dSSc: diffuse systemic sclerosis; SD: standard deviation.

**Table 2:** Characteristics of vascular changes of the eye fundus and the presence of peripheral trophic changes in patients with scleroderma.

Vascular changes of the eye fundus	Number of eyes in SSc patients group			Fingers ulcerations	
	lSSc (*n* = 34)	dSSc (*n* = 16)	SSc (*n* = 50)	Observed (number of patients)	Not observed (number of patients)
AMD (soft drusen and window defects of retinal pigment epithelium)	11	3	14	14	2
Hard exudates	2	4	6	2	1
Irregularities in the course and caliber of the retinal vessels	22	8	30	12	3
Delayed filling of choroidal lobules	14	6	20	9	1
Occlusion of the peripheral arterial vessel	2	0	2	2	0
Sheathed venous vessel	0	1	1	1	0
Thinning of choroidal capillaries and retinal pigment epithelium	4	2	6	3	0
Ischemic retinopathy	2	2	4	1	1
Normal fundus	6	2	8	7	1

Abbreviations: lSSc: limited systemic sclerosis; dSSc: diffuse systemic sclerosis; AMD: age-related macular degeneration.

**Table 3:** Comparison of VEGF parameters between the experimental and control groups.

Parameter	Group		*p* level
	Scleroderma (*n* = 25)	Control (*n* = 25)	
Serum VEGF (pg/ml) (mean ± SD)	346.27 (399.88)	197.737 (155.04)	0.4705
Serum sVEGFR-2 (pg/ml) (mean ± SD)	10184.00 (2507.77)	9110.125 (2858.61)	0.0970
Serum VEGF/sVEGFR-2 (mean ± SD)	0.0775 (0.2373)	0.0350 (0.0752)	0.4081
VEGF in tears (pg/ml) (mean ± SD)	275.30 (552.39)	352.135 (135.25)	<0.001^∗^
sVEGFR-2 in tears (pg/ml) (mean ± SD)	87.25 (69.92)	8274.653 (11033.51)	<0.001^∗^
VEGF/sVEGFR-2 in tears (mean ± SD)	1.1305 (0.7295)	0.2391 (0.3418)	<0.001^∗^

Significant difference according to the Mann–Whitney test. Abbreviations: VEGF: vascular endothelial growth factor; sVEGFR-2: soluble form of vascular endothelial growth factor receptor 2; SD: standard deviation.

**Table 4:** Comparison of VEGF parameters between experimental subgroups and a control group.

Parameter	Group			*p* level
	lSSc (*n* = 17)	dSSc (*n* = 8)	Control (*n* = 25)	
Serum VEGF (pg/ml) (mean ± SD)	384.76 (467.19)	269.30 (218.03)	197.737 (155.04)	0.7348
Serum sVEGFR-2 (pg/ml) (mean ± SD)	10073.03 (3014.4329)	10405.94 (1052.3658)	9110.125 (2858.61)	0.2474
Serum VEGF/sVEGFR-2 (mean ± SD)	0.1032 (0.2898)	0.0260 (0.0226)	0.0350 (0.0752)	0.9515
VEGF in tears (pg/ml) (mean ± SD)	382.86 (672.46)	87.06 (109.45)	352.135 (135.25)	<0.001^a^
sVEGFR-2 in tears (pg/ml) (mean ± SD)	72.68 (61.71)	119.30 (83.35)	8274.653 (11033.51)	<0.001^b^
VEGF/sVEGFR-2 in tears (mean ± SD)	1.1906 (0.5792)	1.0344 (0.9953)	0.2391 (0.3418)	<0.001^c^

^a,b^Significantly lower values of tears VEGF parameters in the lSSc and dSSc subgroups in comparison to a control group according to post hoc analysis of the Kruskal-Wallis ANOVA by ranks. ^c^Significantly higher coefficient of tears VEGF parameters in the lSSc subgroup in comparison to a control group according to post hoc analysis of the Kruskal-Wallis ANOVA by ranks. Abbreviations: VEGF: vascular endothelial growth factor; sVEGFR-2: soluble form of vascular endothelial growth factor receptor 2; lSSc: limited systemic sclerosis; dSSc: diffuse systemic sclerosis; SD: standard deviation.

## Data Availability

The data used to support the findings of this study will be available in excel file with the request to the corresponding author.

## Abbreviations

SSc: Systemic sclerosis
lSSc: Limited systemic sclerosis
dSSc: Diffuse systemic sclerosis
VEGF: Vascular endothelial growth factor
VEGFR-1 membrane receptors (fms-like tyrosine kinase Flt-1): Vascular endothelial growth factor receptor-1
VEGFR-2 (fetal liver kinase-1 Flk, KDR): Vascular endothelial growth factor receptor-2
VEGF-3 (Flt-4, fms-related tyrosine kinase 4): Vascular endothelial growth factor receptor-3
sVEGFR-1: Soluble VEGF receptor 1
sVEGFR-2: Soluble VEGF receptor 2
SD: Standard deviation
ARA: American Rheumatism Association
AMD: Age-related macular degeneration
FA: Fluorescein angiography
ELISA: Enzyme-linked immunosorbent assay.
